# Strengthening health systems: the role of drug shops

**DOI:** 10.1186/s40545-021-00373-0

**Published:** 2021-11-16

**Authors:** Zubin Cyrus Shroff, Nandita Thatte, Shawn Malarcher, Baker Maggwa, Geetanjali Lamba, Zaheer Ud-Din Babar, Abdul Ghaffar

**Affiliations:** 1 Alliance for Health Policy & Systems Research, World Health Organization, Geneva, Switzerland; 2 Implementing Best Practices Network, World Health Organization, Geneva, Switzerland; 3 Office of Population and Reproductive Health, United States Agency for International Development, Bureau for Global Health, Washington DC, USA; 4 Centre for Pharmaceutical Policy and Practice Research, Department of Pharmacy, University of Huddersfield, Queensgate, Huddersfield, UK

## Introduction

Private medicine retailers, or drug shops, are an important source of essential medicines and basic healthcare, particularly in rural areas of low- and middle-income countries (LMICs), where licensed providers are often scarce [1]. Despite the reach and potential role that drug shops play in enabling access to medicines and services among marginalized populations, concerns remain around the quality of services they provide and their regulation, since these often work outside the formal health system. Integrating drug shops into the formal health system has been proposed as a potential way to improve access and health system performance [2]. By recognizing these outlets as an important part of the health system, governments have the opportunity to track their contribution to improved health; create more agile and responsive health markets that are better able to respond to shocks and stressors; and ensure communities served by these vendors receive high-quality products, information, and services [3]. The need for more agile health markets and informed communities with access to high-quality products and services has been greatly underscored by the COVID-19 pandemic. Interventions that strengthen the practice of these shops therefore have the potential to contribute to improved access to and appropriate use of medicines, improved health outcomes and stronger health systems [3]. These interventions are wide-ranging [4]. These include training of drug shop staff, community education, linking drug shops to supply chain mechanisms, enhanced regulation, and the changes in formal policies to enable recognition of drug shops [4–9]. Such diverse interventions influence one or more of the health-system building blocks including financing, health workforce, service delivery and health information systems.

Recognizing the important contribution that drug shops can make in enhancing access to medicines and services in low- and middle-income countries (LMICs), governments have launched several initiatives targeting them over the past decade. These efforts are often focused on a specific health issue; malaria, family planning, HIV, for example and involve one or more of the health systems building blocks described above [5, 6, 10]. To date, there has been limited opportunity for learning across these efforts.

## Program of research

To address the learning gap and better understand how such initiatives have been implemented and have contributed to efforts to strengthen health systems, the Alliance for Health Policy and Systems Research, WHO, Geneva, and the Implementing Best Practices (IBP) Network at WHO, developed a multi-country implementation research program with support from USAID. The objective of the program was to understand the processes and mechanisms through which efforts to engage drug shops in the delivery of specific services have contributed towards strengthening health systems towards universal health coverage (UHC).

Prior to developing the call for implementation research, several activities were conducted to engage NGO, CSO and other implementation partners as part of process. First a robust documentation activity was conducted in several countries (including Uganda and Ghana) to assess the impact and benefits of existing programs focused on provision of services through Drug Shops. Results from these documentation activities were then used to convene a stakeholder workshop in Washington DC in August 2018. This workshop identified challenges and gaps in knowledge faced by program implementers in engaging drug shops toward stronger health systems. This input, taking into consideration gaps from the implementer perspectives, was then used to design the call for implementation research by WHO resulting in a research agenda fully informed by field-based implementation experiences. In addition to known interventions to incorporate drug shops into formal health systems, implementing partners also highlighted issues around drug shop operator motivation, training, and behavior change interventions as potential areas to strengthen integration and sustainability into the health system.

Thirty-seven submissions were received in response to the call for research, which closed in January 2019. Submissions were reviewed by external peer reviewers. Based on this peer review process, seven country teams, from Bangladesh, Indonesia, Myanmar, two teams from Nigeria, Tanzania and Zambia were supported to conduct research. This supplement issue of the *Journal of Pharmaceutical Policy and Practice* presents important findings from the research program. It highlights initiatives implemented in these countries to harness the potential of drug shops in contributing to stronger health systems. In addition to six case studies based on national experiences, it includes one cross-cutting article on factors that make such initiatives successful and one editorial outlining the importance of supply chain management in health systems strengthening efforts. Below we briefly describe each of the articles.

First, Thein et al. examine the role that drug shops play in malaria and tuberculosis programs in Myanmar, where these infectious diseases remain an important cause of morbidity and mortality. Population Services International (PSI) implemented a program for the appropriate screening, referral and management of presumptive TB and malaria cases in Myanmar by drug shops. This initiative involved both a training component, as well as appointing a field supervisor in each township that was able to support both drug sellers and their clients with providing these services. Based on a structured survey instrument, 86.4% of TB providers and 87.7% of malaria providers were found to use the correct criteria for determining a client as a presumptive TB case or malaria case, respectively. An important facilitator to this was the involvement of a field supervisor, who greatly assisted drug shops and their clients. The authors found that barriers to adherence to guidelines included drug shop staff preferring use of their clinical judgement and experience rather than guideline-based screening, referral and management. This demonstrates the potential of drug shops to fully engage with the primary health care system in Myanmar, though the importance of adhering to national guidelines needs to be emphasized [11].

Second, Ferdiana et al. examine the implementation of a public private partnership (PPP) to increase the appropriate use of Artemisinin Combination Therapy (ACT) in the Manokwari district, of West Papua Province, Indonesia, an area where malaria remains endemic. As per the terms of this partnership, private pharmacies are provided ACT at no cost on the condition that these are dispensed based on a physician’s prescription and a positive diagnostic test for malaria. ACT replenishment at the pharmacies is contingent on their providing evidence of adherence to dispensing guidelines. The initiative was associated with a substantial increase in the proportion of malaria cases in the district reported by private pharmacies (from 6.9% in 2018 to over 30% in 2019). Pharmacies also reported that the PPP had improved the consistency of ACT supply. However, the coverage of the PPP remained limited with a relatively small proportion of pharmacies in the district actively engaged in the initiative. Reported reasons for this included the limited involvement of physicians in the initiative (some of whom continued to prescribe ACTs without a diagnostic test), increased administrative burden on pharmacies around data reporting and drug procurement, and the lack of financial incentives [12].

Patent medicine vendors (PMVs) are a major source of contraception in Nigeria due to geographical, financial and sociocultural factors, and injectable contraception remains a popular choice, accounting for approximately 30% of all contraception used by women in Nigeria. Oluwasanu et al. explore the processes and mechanisms through which a pilot intervention to train PMV staff to administer injectable contraceptives increased women’s utilization of these products. Based on interviews of PMVs, users, as well as other key-stakeholders employing a mixed methods design, they found that the training PMVs received as part of the pilot intervention and a positive regulatory environment that was enabled by the government temporarily allowing PMVs to legally provide injectable contraceptives as part of the pilot intervention were enablers to increasing the uptake of injectable contraception. On the other hand, ensuring consistent supply of high-quality injectable contraceptives was a challenge. Referral and linkages between PMVs and other facilities that was an integral part of the intervention remained sub-optimal as did PMV maintenance of records of services delivered [13].

Also in Nigeria, Uneke et al. conducted an assessment of access to patent and proprietary medicine vendors (PPMVs) among nursing mothers and young people in rural communities of south-eastern Nigeria. Using structured questionnaire tools to assess client perceptions of PPMV services and access to medicines among rural populations, they found that nursing mothers overwhelmingly treated children’s coughs and cold with antibiotics without consulting a health worker. Respondents largely agreed with the statement that the sale of medicines without a prescription was rampant in their locality. PPMVs were the source of a majority of antibiotics used. Among youth in the area, there was general agreement that PPMVs were easily accessible as a source of care for the rural population. However, only a little over a quarter of youth respondents reported using PPMVs to purchase family planning products, with over 95% of youth on the other hand reporting that they had used PPMVs as a source of antimalarial drugs. The authors highlight the need to train PPMVs in order to enable them to legally provide antibiotics and contraceptives within rural communities, also underscoring the importance of policy change to this end [14].

Next, the paper by Zulu et al. from Zambia examines the implementation of guidelines by that country’s medicine regulatory agency to put in place dispensing facilities known as health shops to increase access to quality medicines, particularly in rural areas. These health shops are authorized to sell a defined list of medicines, including anti-malarials and oral contraceptives, over the counter. The guidelines include standards around human resources, drug supplies, waste disposal and record keeping facilities. Factors that facilitated acceptability of guidelines included their perceived relevance, comprehensive training on the guidelines, as well as the potential of their implementation to improve overall delivery of services and enhance the status of these shops within the community. On the other hand, their high cost of implementation, including through what were perceived to be onerous registration requirements as well as the restrictions the guidelines imposed on the medicines that they could sell, were viewed as deterrents to participation in the initiative. The study found that the implementation of the guidelines was associated with the ability of health shops to provide a range of informational and diagnostic services as well as increased referral to public facilities, demonstrating their potential to contribute to stronger health systems in rural Zambia [15].

Bangladesh’s National Drug Policy forbids antibiotic sale in the absence of a physician’s prescription. However, antibiotics continue to be available over the counter, with negative implications for the emergence of anti-microbial resistance. Nizame et al. explore awareness of the policy among private drug shop operators as well as users of these shops to identify barriers and facilitators to compliance with the policy. Barriers to compliance with the policy included a limited understanding of the policy among drug shops, as well as disagreements with the content of the policy; despite drug shop operators not being able to legally prescribe antibiotics, many of them felt that they should have the right to do so. In addition, appropriate antibiotic prescribing was hampered by a shortage of qualified physicians [16].

The final paper based on the country studies by Lamba et al. brings together learning from the seven projects supported by this research program to better understand the range of factors and their interactions that explain the success of initiatives developed by governments to engage drug shops towards stronger health systems in these settings. The authors use a modified version of Bigdeli et al.’s framework on access to medicines to identify factors at the micro (individual), meso (health service delivery) and macro (health sector and national context) levels. At the micro-level, the fact that drug shops were often the first and preferred source of care, as well as good pre-existing relationships between community members and drug shop users were found to be important determinants of initiative success. At the level of service delivery, client pressure to provide drugs on demand, which was nearly universal across the countries studied, challenges around procurement, as well as financial and administrative burdens related to the initiative (including around reporting requirements) were seen to negatively influence perceived success. At the macro-level, stakeholder buy-in from multiple sectors and different levels of the health system was identified as a key factor to initiative success. The importance of the different factors varied across settings, with initiative success often dependent on how factors interacted with each other. The paper concludes with recommendations that emphasize the importance of financial incentives aligned with public health goals as well as multi-stakeholder collaboration that is often central to policy and legal changes to enable drug shops to contribute to sustainable systems change [17].

To conclude the supplement, an editorial by Ghaffar et al. articulates the importance of supply chain management in access to medicines and health services in low- and middle-income countries (LMICs). The COVID-19 pandemic has highlighted the significance of supply chain management of essential medicines and services, including therapeutics, diagnostics and vaccinations. The authors make the case that supply chains are an intrinsic part of the health system and that supply chain management should be recognized as an essential competency for public health professionals. This thus calls for the immediate attention of academics, funders and policymakers towards the subject of supply chain management, including through supporting formal training of practitioners in this area [18].

## Conclusion

The articles in this special issue offer new knowledge on how countries have been able to implement initiatives engaging drug shops in a variety of settings and targeting a broad range of issues. They demonstrate the importance of initiatives that combine efforts to regulate drug shops with tangible incentives (whether they are financial incentives or incentives in terms of recognition) for drug shops to engage with the formal health system. They make clear the central role of people in both the success of initiatives as well as their failure. The ease of access to drug shops in rural areas is what enables them to contribute to service delivery in places where there are few if any formal providers. However, in almost all studies, drug shops noted that client pressure to provide medication on demand was a major barrier to efforts to ensure that access to medicines was appropriate, something that is central to preventing misuse of antibiotics. The studies also make the case for greater engagement with drug shop operators in the development of policies and programs that affect them. This will enable an increased sensitization to the public health challenges resulting from unrestricted access to medicines as well as the co-production of policies and guidelines that have the buy-in of drug shops and that can be feasibly implemented in these settings. By engaging implementers in the generation of research priorities and framing research questions, the program has resulted in the production of findings that are directly relevant to real-world program implementation and may contribute to longer term sustainability once fully implemented. Additionally, this program has also strengthened research capacities within the study countries, enlarging the community of researchers engaged in the study of more effectively using drug shops to contribute to UHC objectives.

Finally, the ongoing collaboration between the Alliance, the Implementing Best Practices Network and the country-based research teams demonstrates the Alliance for HPSR’s unique role in convening diverse groups of actors and organizations. Together, the new knowledge generated by this group can help inform approaches for the design and implementation of successful strategies to engage drug shops to bring about sustainable systems change to move towards UHC.

## Data Availability

Not applicable.
